# Up-regulation of A20/ABIN1 contributes to inefficient M1 macrophage polarization during Hepatitis C virus infection

**DOI:** 10.1186/s12985-015-0379-0

**Published:** 2015-09-17

**Authors:** Chao Fan, Ying Zhang, Yun Zhou, Bingjie Li, Yu He, Yonghong Guo, Zhansheng Jia

**Affiliations:** Department of Infectious Diseases and Center of liver Diseases, Tangdu Hospital, the Fourth Military Medical University, Xi’an, 710038 China

## Abstract

**Background:**

Anti-hepatitis C virus (HCV) responses are often accompanied by an increase in alanine aminotransferase levels in HCV-infected patients, indicating that inflammatory responses are compromised by the virus. Additionally, inflammation is associated with M1-polarizated macrophages, which secrete cytokines such as tumor necrosis factor-α, interleukin-1, and interleukin-12, and present antigens through phagocytosis. HCV-encoded proteins are presented as specific viral antigens in particular infectious steps that influence the immune response. For instance, HCV antigens impact macrophage PD-1 and Tim-3 expression, and contribute to impaired viral clearance. Furthermore, circulatory HCV antigens from infected patients inhibit dendritic cell differentiation, which raises the possibility that HCV antigens may also interfere with macrophage polarization.

**Methods:**

In this study, the impact of HCV antigen stimulation on M1-polarized macrophages was investigated. The influence of HCV antigens was evaluated by reverse transcription polymerase chain reaction and enzyme-linked immunosorbent assay. Specific changes were investigated clinically by flow cytometry and immunofluorescence. Effects of NF-κB during the process were analyzed by western blot.

**Results:**

HCV infection dampened M1 macrophage polarization *ex vivo* and *in vitro*. After antigen stimulation, NF-κB signaling was suppressed by the up-regulation of A20 and A20-binding inhibitor of NF-κB binding protein, which likely leads to a variation of functional molecules such as tumor necrosis factor-α, CD163, matrix metalloproteinases, transferrin receptor-1, and CD100, reflecting an anti-inflammatory reaction against M1-polarization.

**Conclusion:**

HCV antigens stimulation up-regulates A20/A20-binding inhibitor of NF-κB binding protein expression, which consequently contributes to inefficient M1 macrophage polarization.

**Electronic supplementary material:**

The online version of this article (doi:10.1186/s12985-015-0379-0) contains supplementary material, which is available to authorized users.

## Background

Hepatitis C virus (HCV) accounts for millions of viral hepatitis cases each year [1]. Because most hepatitis symptoms are mild or even absent, patients often fail to report chronic infection later in life. Anti-HCV responses are often accompanied by an increase in alanine aminotransferase (ALT) levels. Patients exhibit persistent mild up-regulated ALT in chronic HCV, indicating that the virus may dampen inflammatory responses. Inflammation is associated with professional antigen presenting cells (APCs) including dendritic cells (DCs) and macrophages that secrete cytokines such as tumor necrosis (TNF)-α and interleukin (IL)-12, and present antigens for T and B cells to facilitate immune responses [2, 3].

Macrophage-augmented inflammatory responses are associated with their differentiation and polarization. Monocytes differentiate to act both as phagocytes and APCs, and are involved in the clearance of pathogen-derived particles and toxins. Myeloid-derived monocytes migrate to different tissues through a C-C chemokine receptor 2-initiated process, and then differentiate into unique macrophages [4, 5]. Two primary macrophage subgroups have been proposed, termed classically activated macrophages (M1) and alternatively activated macrophages (M2). The M1 phenotype is characterized by IL-12^hi^IL-23^hi^IL-10^lo^ expression and iron uptake activity, facilitating inflammatory, microbicidal, and tumoricidal functions. M2 macrophages express IL-12^lo^IL-10^hi^IL-1decoyR^hi^ IL-1RA^hi^ and release iron, inhibiting inflammation, and promoting tissue remodeling and parasite clearance [6–8].

In chronic hepatitis C (CHC), HCV antigen stimulation leads to profound changes in host immune cells. For example, natural killer (NK) cells are directly inhibited by exposure to HCV E2 protein [9–11], while HCV core protein peptides facilitate HLA-E expression and thereby impair NK cell cytotoxicity [12, 13]. HCV core and NS3 antigens activate monocytes to produce IL-10 and TNF-α, as well as suppress IL-29 secretion, causing a functional inhibition of DCs [14, 15], and facilitating dimerization of Toll-like receptors (TLR) 1/2 and 2/6 and hepatic fibrogenesis [16–19]. Recent experiments indicated that HCV present in the sera of infected patients interferes with DC differentiation *in vitro* [20], indicating that such antigens may also interfere with macrophage function during differentiation.

Studies have shown that HCV antigens influence macrophage responses and cytokine expression, alter signaling receptors and adhesion molecule levels [21–23]. HCV also influences DC function by modulating their maturation and cytokine production [24]. Macrophage TLR-3, PD-1, and Tim-3 expression mediate HCV infection, leading to dysfunctional macrophage function [25, 26]. However, whether HCV impacts macrophage polarization is still poorly understood.

HCV antigens affect macrophages through various signaling molecules. In this study we analyzed TNF-α, IL-10, IL-12, transferrin receptor-1 (TfR1), and matrix metalloproteinases (MMPs) that are highly associated with macrophage polarization. We examined monocytes from healthy donors and HCV patients that were isolated and treated with IFN-γ/lipopolysaccharide (LPS) to stimulate M1 polarization *ex vivo*. The data indicated that macrophages from HCV infected patients exhibited decreased TNF-α and TfR1 expression levels. HCV antigen stimulation of macrophages altered gene expression associated with M1 polarization, including the expression of TNF-α, IL-10, IL-12, CD163, TfR1, MMPs and CD100, as well as inhibition of macrophage phagocytosis of HCV in cell culture (HCVcc). Furthermore, NF-κB activity was abnormally suppressed and A20 with its binding partner A20-binding inhibitor of NF-κB binding protein (ABIN1) was up regulated after polarization. A20/ABIN1 overexpression had a similar influence on M1 polarization compared with HCV antigen stimulation. As the A20/ABIN1 complex negatively regulates NF-κB signaling [27, 28], HCV-induced A20/ABIN1 expression likely inhibits NF-κB and contributes to abnormal M1-polarization.

## Results

### Macrophages from HCV-infected patients are polarized abnormally *ex vivo*

**Fig. 1:**
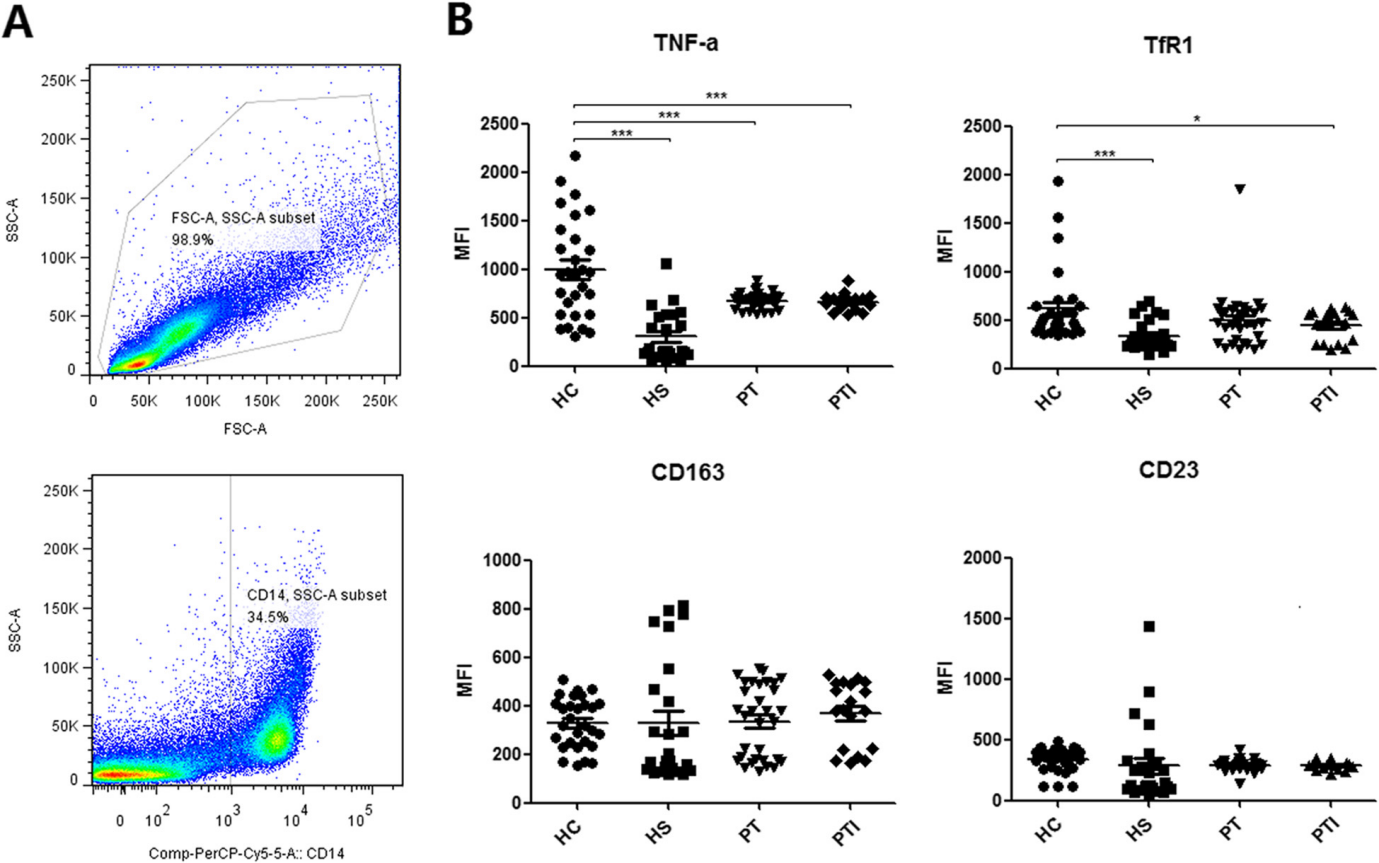
Macrophage phenotypic changes after HCV antigen exposure. Monocytes from healthy donors (HC), healthy donors with HCV antigen stimulation (HS), treated patients (PT) and patients with liver injury (PTI) were treated with LPS/IFN-γ for polarization before analysis. (**a**) Gating strategy. (**b**) Mean fluorescence intensity (MFI) of TNF-α, TfR1, CD163 and CD23 expression on macrophages. One-way ANOVA was used to determine statistical significance (*p < 0.05, ***p < 0.001)

**Fig. 2:**
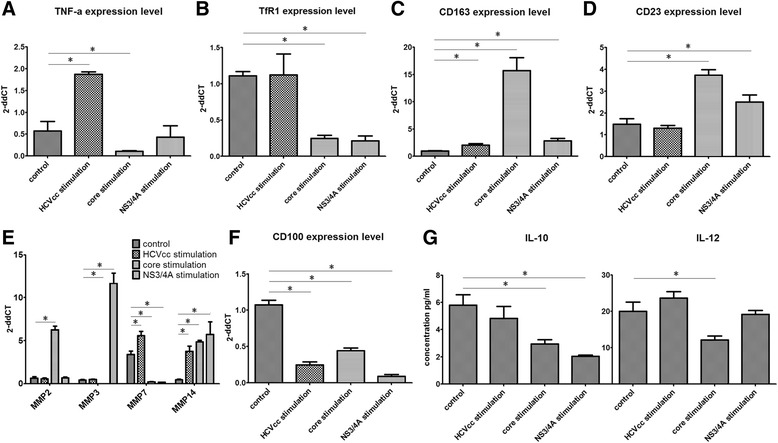
HCV antigen pre-stimulation influences macrophage polarization *in vitro*. THP-1 cells were incubated with or without HCV antigens (HCVcc, core protein or NS3/4A) after PMA stimulated macrophage differentiation. Adherent cells were then treated with LPS/IFN-γ medium for M1-polarization before analysis. TNF-α (**a**), TfR1 (**b**), CD163 (**c**), CD23 (**d**), MMPs (**e**) and CD100 (**f**) expression levels were measured by RT-PCR. IL-10 and IL-12 expression levels from the culture medium were measured by ELISA (**g**)

Peripheral blood mononuclear cells (PBMC) from healthy donors (*n* = 29) were cultured with normal medium (HC group) or stimulation medium (HS group, containing HCV-core protein and NS3/4A) for 1 day and were then treated with LPS/interferon (IFN)-γ for M1 polarization. Macrophages were then isolated and analyzed by flow cytometry (Fig. 1a). As expected in response to LPS/IFN-γ challenge, M1 polarization stimulated inflammatory cytokines (TNF-α and IL-12) [29–31], and TfR1 iron uptake receptors, and down-regulated scavenging receptors (MARCO, CD163, and CD23) [32–34]. However, compared with normal polarization, HCV antigen stimulation reduced TNF-α and TfR1 expression (*p* < 0.001), suggesting suppression of M1 polarization (Fig. 2b). Although CD163 and CD23 expression was not significantly different, a slight up-regulation was observed in some cases.

Macrophages from CHC patients receiving antiviral therapy (PT: *n* = 32) were also tested. The patients were further divided into response and no response groups by alanine aminotransferase (ALT) levels (PTI group: ALT > 40U/L). Patient PBMCs were cultured with normal medium for 1 day prior to LPS/IFN-γ M1 polarization. Macrophages isolated and analyzed by flow cytometry showed decreased TNF-α and TfR1 levels (PT: TNF-α *p* < 0.001, PTI: TNF-α *p* < 0.001 and PTI: TfR1 *p* < 0.05) (Fig. 1b), indicating impairment of M1-polarization. Nevertheless, CD163 and CD23 did not show much variation, which may indicate more complex cell–cell interactions *in vivo*.

### HCV antigens influence macrophage polarization by different mechanisms *in vitro*

The impact of HCV antigens on M1 polarization was investigated using the THP-1 monocyte/macrophage system. TNF-α, TfR1, CD163, CD23 and MMP expression levels were tested. The HCVcc and core protein showed different effects on TNF-α expression, as HCVcc enhanced TNF-α expression and the core protein reduced TNF-α expression (Fig. 2a). Nevertheless, the main effects of HCV antigens should be TNF-α inhibition according to *ex vivo* data that showed a decreasing trend in the patient group. An inhibitory effect was observed for TfR1 (Fig. 2b and Additional file 1: Figure S1). TfR1 mediates iron endocytosis, but decreased TfR1 expression resulting from HCV antigen stimulation blocks M1 iron uptake activity. CD163 and CD23 are important macrophage-specific receptors that switch between activated phenotypes during inflammation [35]. Although there was only a small change in CD163 and CD23 expression *ex vivo*, both were increased after incubation with HCV antigens (Fig. 2c, d) *in vitro*. M1 macrophages are characterized by high expression levels of TNF-α and TfR1, as well as low expression levels of CD163 and CD23. The variation of these molecules may have a long-term effect on the polarization process.

MMPs generally control extracellular matrix (ECM) remodeling and have extensive influence on macrophage migration and cell metabolism [36]. Our data revealed that HCV core protein induced MMP2 expression, while HCVcc and NS3/4A had little effect. MMP3 expression decreased after core protein treatment and increased in NS3/4A-stimulated cells, while MMP7 expression inhibited HCV core protein and NS3/4A, and HCVcc stimulated its expression. Interestingly, MMP14 expression was elevated by all HCV antigens, which represents nonspecific changes in response to exogenous substances (Fig. 2e).

We next asked whether HCV stimulated expression of MMP14 target proteins. MMP14 digests CD100 from the macrophage cell surface to produce an essential source of soluble CD100 (sCD100) [37], which is a universal activator of immune cells. CD100 elicits T and B lymphocytes and NK cells by inhibiting the inhibitory receptor CD72, leading to the activation of target cells. sCD100 in particular detaches from the membrane and diffuses into the blood, and has greater inflammatory activity than its membrane form [38, 39]. However, although increased MMP14 expression increases sCD100 levels, total macrophage CD100 expression was restrained after HCV core and NS3/4A stimulation, suggesting extensive immune suppression by HCV antigens (Fig. 2f).

IL-10 and IL-12 expression levels were also altered in response to different HCV antigenic stimulation, indicating that abnormal polarization maybe induced by different antigens (Fig. 2g). Interestingly, IL-10 secretion was decreased following incubation with HCV antigens, implying a more significant M1-polarization (especially for the NS3/4A group), whereas HCV core stimulation reduced IL-12 secretion after polarization, suggesting impairment of M1-polarization. Macrophages are the primary source of IL-12 involved in Th1 and Th17 responses, and increased IL-12 expression is an important characteristic of M1 polarization [6, 40, 41]. Thus, a decrease in IL-12 secretion caused by HCV core antigens likely dampens M1-induced inflammation.

### HCV antigen pre-incubation influences A20/ABIN1 complex in M1 macrophages

**Fig. 3:**
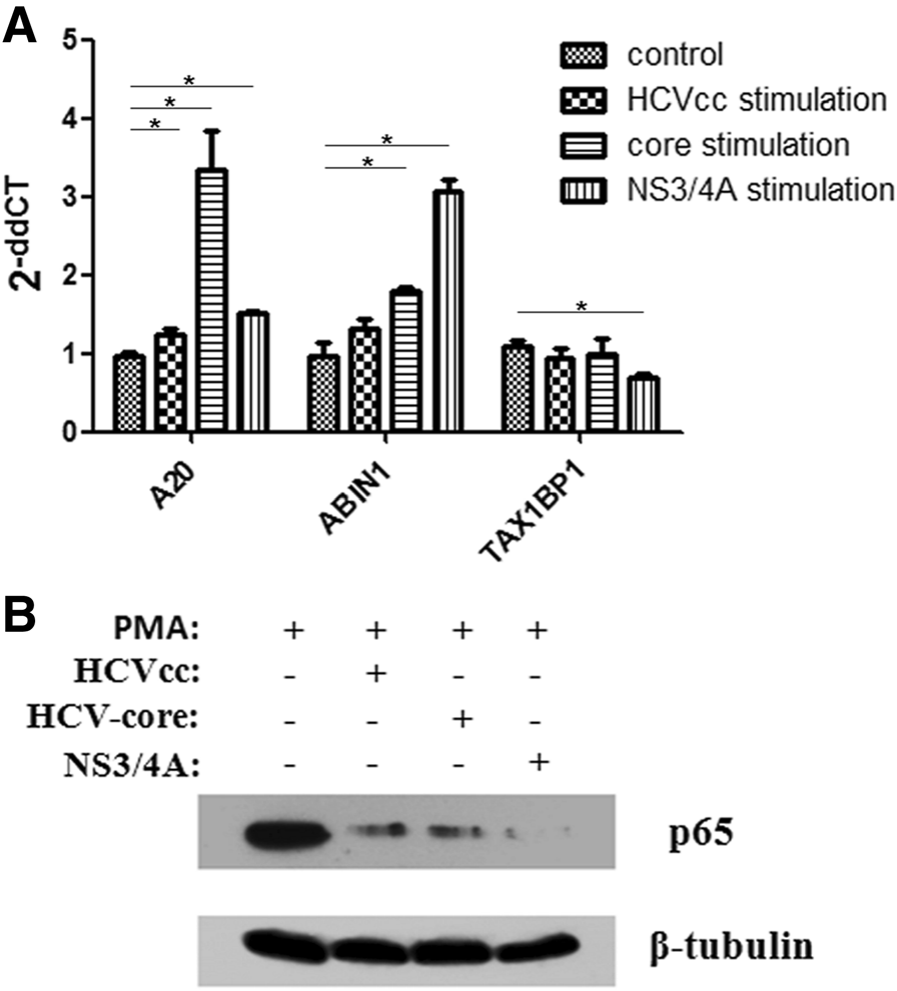
HCV antigen stimulation suppresses NF-κB activity in polarized M1 macrophages. **a** The expressions of A20/ABIN1/TAX1BP1 were distinctively influenced by each antigen. Core protein highly increases A20 levels and NS3/4A enhances ABIN1 expression. TAX1BP1 shows a slight reduction trend by NS3/4A stimulation. **b** NF-κB activity was down regulated by HCV antigen stimulation, with regard to p65 phosphorylation

Various signaling pathways influence macrophage maturation, including STATS [42–46], MAPK [47–49], and NF-κB [50, 51]. TLR engagement induces NF-κB activity, producing inflammatory mediators associated with M1 polarization. Conversely, M1 related gene suppression is commonly associated with blockage of NF-κB co-activators [52]. Our experiments tested NF-κB activity in each group. Compared with normal M1 polarized cells, NF-κB signaling occurred at a lower level (p65 phosphorylation) when pre-incubated with HCV antigens (Fig. 3b). We further checked expression of the A20/ABIN1/TAX1BP1 complex, which regulates NF-κB activity. The data suggest that expression was distinctively influenced by each antigen (Fig. 3a). The HCV core protein significantly enhanced A20 expression, while HCVcc and NS3/4A contributed modestly. ABIN1 was up regulated by all antigens, while NS3/4A contributed more to efficiency. However, TAX1BP1 showed a slight reduction, especially when stimulated by NS3/4A. As the A20/ABIN1 complex suppresses NF-κB through the regulation of ubiquitin, which targets TBK1/IKKi, the total effect of HCV antigen stimulation should weaken NF-κB activity [28].

### Enhanced A20/ABIN1 contributes to abnormal M1 polarization

**Fig. 4:**
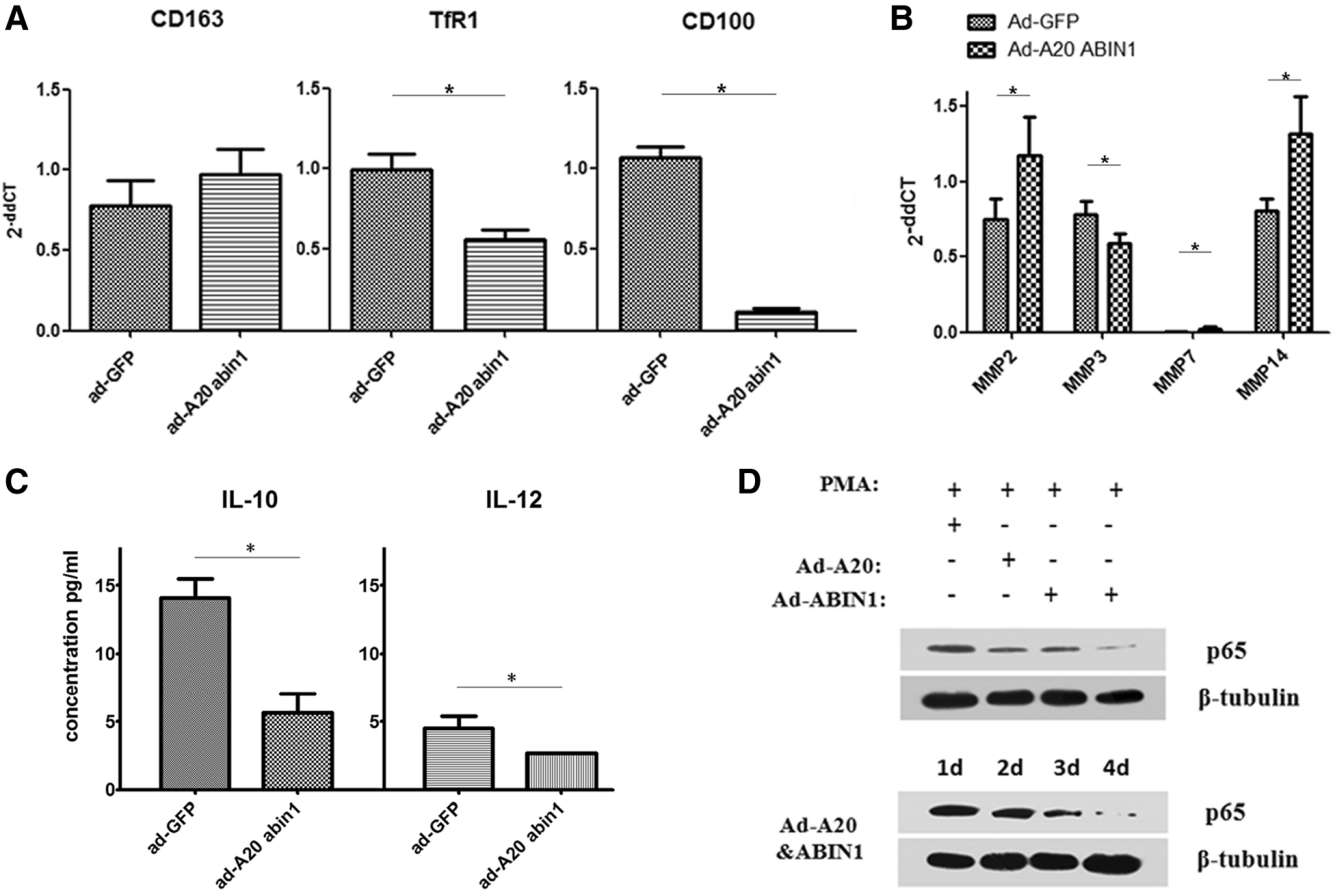
A20/ABIN1 overexpression contributes to abnormal M1 polarization. A20 and ABIN1 were over expressed in THP-1 cells (incubated with LPS/IFN-γ before analysis) during PMA stimulation. **a** A20/ABIN1 over-expression reduced CD100 and TfR1 expression and CD163 was slightly up regulated. **b** A20/ABIN1 over-expression influences MMPs expression differently. **c** IL-10 and IL-12 were decreased by A20/ABIN1 over-expression. **d** Over-expression of A20 or ABIN1 inhibited NF-κB activity, and co-expression had a greater efficacy (*upper*). NF-κB activity was gradually reduced at 3 days incubation (*lower*)

We next determined whether overexpression of A20 and ABIN1 during macrophage differentiation induced similar effects to HCV antigen stimulation. A20/ABIN1 were overexpressed by adenovirus vectors in THP-1 cells and stimulated with phorbol myristate acetate (PMA). The expression efficiency was checked by co-expression marker green fluorescent protein (GFP) (Additional file 2: Figure S4). The overexpression of A20 and ABIN1 each inhibited NF-κB activity, as expected, whereas co-expression of the two proteins was more efficient. NF-κB activity gradually reduced following 3-day PMA stimulation (Fig. 4d). CD100, TfR1, IL-10, and IL-12 expression declined, while CD163 increased after A20/ABIN1 overexpression, correlating with HCV antigen stimulation (Fig. 4a, c). The influence on MMPs appears more complicated, as core MMP2 and MMP3 antigens, and MMP7 in the HCVcc group and MMP14 in all HCV antigens, showed a similar response to A20/ABIN1 overexpression, indicative of distinct regulatory mechanisms (Fig. 4b).

M2 inhibition of NF-κB/HIF-1α–dependent gene transcription is dependent upon STAT3/STAT6 activation [8]. During macrophage polarization, if A20/ABIN1 is overexpressed, NF-κB may be suppressed because of a negative A20/ABIN1 feedback loop, which may have profound influences on polarized macrophages. During HCV infection, HCV antigens exist in a number of organs as well as peripheral blood [53], suggesting that HCV stimulates A20/ABIN1-mediated inflammation via macrophages.

### A20/ABIN1 up-regulation dampens HCVcc phagocytosis of M1 macrophages

**Fig. 5:**
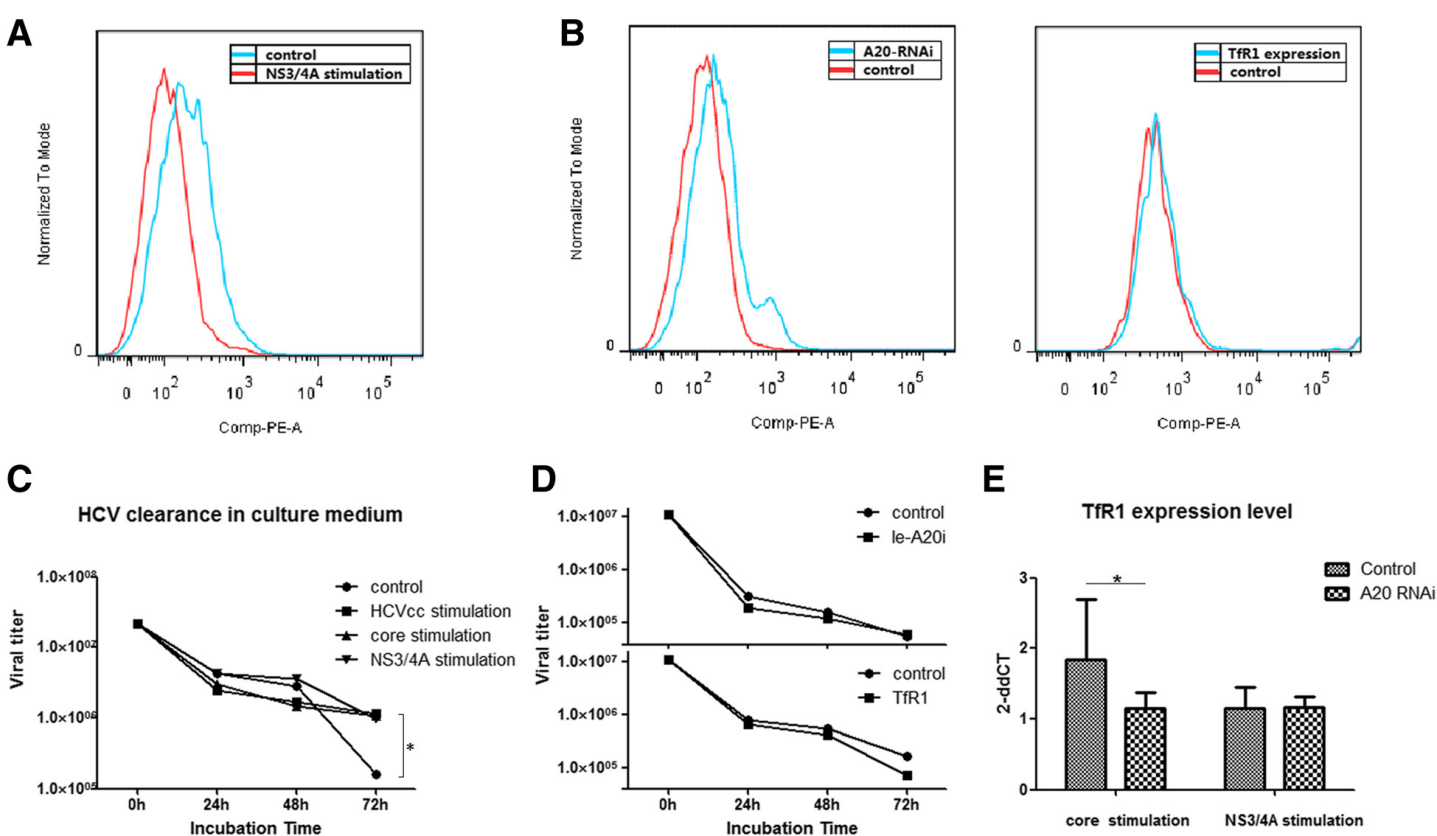
A20 and TfR1 affect macrophage phagocytosis activity differently. **a** NS3/4A stimulated macrophages endocytosed fewer HCVcc particles. **b** A20 suppression by RNA interference rescues phagocytosis activity. In contrast, TfR1 over-expression did not rescue phagocytosis. **c** Viral titers in culture medium from HCV antigens stimulation were significantly higher than normal controls (*p* < 0.001). **d** TfR1 over-expression, but not A20 knockdown, reduces viral load extracellularly. **e** A20 knockdown cannot rescue TfR1 expression at the transcription level

As professional APCs, macrophages not only mediate nonspecific responses against microbes, but also induce antigen-specific immune responses through antigen presentation. Macrophages present antigens that initiate B cell activation. Phagocytosis plays an essential role in this and is the first step of antigen presentation [54]. To examine whether premature macrophage–HCV antigen interaction impacts phagocytosis, stimulated THP-1 cells with or without HCV-NS3/4A co-incubation were exposed to HCVcc for 6 hours. Cells were harvested and analyzed by flow cytometry. The results indicated that macrophages exposed to NS3/4A generated fewer HCVcc particles, suggesting reduced HCV phagocytosis (Fig. 5a and Additional file 3: Figure S2). Because HCV antigens stimulate A20/ABIN1 expression, we further suppressed A20 by RNAi during stimulation and examined M1 phagocytosis activity by flow cytometry. Interestingly, an enhanced expression peak emerged, which suggests macrophage phagocytosis was rescued by A20 knockdown, indicating that A20 up-regulation contributes to reduced phagocytosis (Fig. 5b and Additional file 4: Figure S3).

We next asked whether this reduction influenced viral concentrations in the ECM, which may be related to viral clearance. The culture medium viral titers were tested at each time point and after 72 hours co-incubation. HCVcc levels in antigen-exposed cells were significantly higher than normal controls (*p* < 0.001), suggesting that HCV clearance from ECM may be impaired (Fig. 5c). However, the extracellular medium viral titer did not change after A20 knockdown, suggesting that phagocytosis activity and ultimate ECM viral concentration may be determined by different mechanisms (Fig. 5d, *upper*).

### A20/ABIN1-mediated TfR1 reduction of macrophages is associated with extracellular HCVcc concentrations

TfR1 is ubiquitously expressed in most tissues, connecting the extracellular ironbound transferrin and endocytic adaptor TTP to facilitate clathrin-dependent endocytosis. Recent studies have shown that TfR1, besides its iron balance function, also participates in HCV entry of infected cells. As the A20/ABIN1 complex inhibits NF-κB signaling, which in turn regulates TfR1 expression through HIF-1 regulation, the change of HCV entry in M1 macrophages may be caused by a variation in TfR1 expression [55]. However, TfR1 overexpression does not rescue HCVcc phagocytosis from NS3/4A stimulated macrophages (Fig. 5d, *lower*). Although macrophage phagocytosis was rescued in A20 RNAi assays, the TfR1 expression level did not increase (Fig. 5e), suggesting that the macrophage phagocytosis process is influenced by A20/ABIN1 and is independent of a TfR1 mediated process. Interestingly, although TfR1 does not play a role in intracellular HCV endocytosis, it reduced the final extracellular concentration, as TfR1 overexpression decreased ECM viral titer, indicating that TfR1 likely mediates the active transport of HCVcc particles.

## Discussion

Our study demonstrated that HCV-infected patients possess functionally defective M1 macrophages. Decreases in TNF-α and TfR1 have positive effects on immune cell activation and iron uptake activity *ex vivo*, indicating suppression of the M1-inflammatory response. Furthermore, *in vitro* data indicated that HCV antigen exposure to monocytes influenced M1-polarization by decreasing IL-12, CD100 and TfR1, as well as up-regulating CD163 and MMPs, implying compromised cell polarization. Thus, HCV antigen stimulation seems to inhibit inflammatory cytokines and iron uptake activity, facilitate anti-inflammatory receptor expression; taken together this characterizes an M1-polarization antagonized phenotype. Notably, rather than inducing macrophage transformation from M1 to M2, HCV stimulation caused a dysfunction of the inflammatory process which both impair M1 and M2 polarization, regarding to weakened phagocytosis activity (Fig. 5) which would be enhanced in transformation of M1 to M2 [6–8].

The liver contains large numbers of tissue-resident macrophages, Kupffer cells, which might be impacted similarly. Kupffer cells contribute to immune regulation in the liver. They express immune sensing receptors, through which they contribute to inflammatory induction in the liver by releasing pro-inflammatory mediators [56]. At the same time, they also express potent anti-inflammatory cytokines and contribute to the local regulation of innate and adaptive immune responses [57–60]. HCV antigen stimulation induces anti-inflammatory processes that contribute to chronic HCV infection in the liver.

HCV-infected patients often exhibit insufficient immune responses in the clinic. During the early stages of infection, circulating HCV RNA is detected 3–7 days after exposure, but seroconversion occurs weeks or months after infection, implying HCV immune suppressive effects. Indeed, humans possess a negative feedback loop in response to inflammation. However, in HCV infection, this negative response may be too sensitive to stimulate an efficient inflammatory response, which requires extra drugs such as PEG-IFN-α. According to our data, the exposure of HCV antigens on monocytes probably contributes to anti-inflammatory processes during HCV infection.

To further interpret the mechanism of HCV antigen induced macrophage variation, NF-κB activity was analyzed. NF-κB signaling plays an essential role in immune regulation and macrophage polarization [55]. Following HCV antigen stimulation, M1-polarized macrophages exhibited an A20/ABIN1 up-regulated phenotype, which suppresses NF-κB activity. Antigen stimulation was antigen-specific, as HCV core protein increased A20, whereas NS3/4A up-regulatedABIN1. As expected, overexpression of A20/ABIN1 generated a similar phenotype to that by HCV antigen stimulation, including decreased CD100, TfR1, IL-10 and IL-12 expression, and increased CD163 and MMP14 expression, as well as reduced phagocytosis. Summarily, A20/ABIN1 up-regulation induced by HCV antigens suppressed NF-κB activity and influenced macrophage differentiation.

Interestingly, A20 knockdown rescued phagocytosis, but had less effect on medium virus concentrations, whereas TfR1 overexpression reduced extracellular viral load. The iron regulator TfR1 is an important HCV entry factor [55]; thus, TfR1 may be involved in macrophage-mediated HCV phagocytosis, or may impact HCV clearance via the ECM.

## Materials and methods

### Subjects

Study subject information is summarized in Additional file 5: Table S1. The first group comprised 30 female healthy donors, the second group included 7 HCV-infected patients who were untreated (treatment-naive patients), and the third group included 32 patients who had been undergoing IFN-α/ribavarin antiviral therapy for 1–6 months (patients receiving antiviral treatment, PT). All patients and healthy donors were negative for HIV and hepatitis B virus. Testing of ALT and HCV viral load was performed by the Department of Infectious Diseases at Tangdu Hospital. The protocol conformed to the ethical guidelines of 1975 Declaration of Helsinki and was approved by the Research and Ethical Committee of Tangdu Hospital of the Fourth Military Medical University. Informed consent was obtained from all donors.

### Cells and stimulation

PBMCs were isolated from blood with Ficoll-Hypaque density centrifugation (Sigma, St Louis, MO, USA) according to the manufacturer’s protocol. Healthy donors PBMCs were cultured with X-vivo 15 medium (Lonza, Walkersville, MD, USA) or stimulation medium (X-vivo 15 containing HCV-core protein and NS3/4A, American Research Products Inc. Waltham, MA, USA) for 1 day and were then treated with LPS/IFN-γ for M1 polarization. Patient PBMCs were cultured with X-vivo 15 medium for 1 day prior to LPS/IFN-γ M1 polarization.

THP-1 cells cultured in RPMI 1640 (Mediatech Inc, Manassas, VA, USA) were obtained from ATCC (Manassas, VA, USA). Huh7.5 human hepatoma cells (ATCC) were grown in Dulbecco’s Modified Eagle’s Medium (Mediatech Inc.) containing 10 % fetal bovine serum (Life Technologies, Grand Island, NY, USA), 100 μg/ml penicillin–streptomycin, 0.05 mM β-mercaptoethanol and 2 mM L-glutamine (Mediatech Inc.). Cells were cultured at 37 °C with 5 % CO_2_. For cell stimulation, THP1 cells were cultured for 3 days in 1640 medium with PMA for differentiation [61, 62] with or without HCV-core, NS3/4A (American Research Products Inc. Waltham, MA, USA) or HCVcc. Cells were then cultured in RPMI 1640 containing IFN-γ/LPS for M1 polarization [63–65] 1 day before analysis.

### HCVcc generation and vector construction

HCVcc was generated by transfection of the HCV pJFH1 transcript (generated by TranscriptAid T7 High Yield Transcription kit, ThermoFisher Scientific, Waltham, MA, US) into Hu7.5 cells using DMRIE-C reagents (Invitrogen, Carlsbad, CA, USA). A20 and ABIN1 adenovirus (ad-A20 and ad-ABIN1), A20 RNAi lentiviral (le-A20i), and TfR1 (pGV146-tfr1) overexpression vectors were purchased from Genechem Co. Ltd (Shanghai, China).

### Western blotting

Cells were harvested in lysis buffer (50 mM Tris–HCl at pH 7.5, 150 mM NaCl, 2 mM EDTA) supplemented with protease inhibitor (Roche Applied Science, Indianapolis, IN, USA). Lysate was run by 10 % sodium dodecyl sulfate polyacrylamide gel electrophoresis and transferred to polyvinylidene difluoride membranes (Immobilon-P, Millipore, Billerica, MA, USA). Membranes were blocked with 5 % (wt/vol) non-fat milk followed by incubation with primary antibodies (p-NF-κB p65 mouse mAb, Cell Signaling, Danvers, MA, USA) and secondary antibody (horseradish peroxidase-conjugated goat anti-mouse, Abgent, San Diego, CA, USA). Membranes probed with β-actin monoclonal antibody (Bioworld Technology, St. Louis Park, MN, USA) served as a control.

### Immunofluorescence

Cells in chamber slide wells were fixed with paraformaldehyde and stained as follows. Primary antibodies against TfR1 (eBioscience, San Diego, CA, USA) and HCV core (mAb to HCV core Antigen, Abcam, Cambridge, MA, USA) were incubated at a 1:2000 dilution overnight at 4 °C. Conjugated secondary antibodies (anti-mouse Alexa-555, Abcam) were incubated at a 1:1000 dilution for 1 h at room temperature. Nuclei were stained with 4′,6-diamidino-2-phenylindole (DAPI). Images were captured via confocal microscopy (BX51-DP70 system, Olympus, Tokyo, Japan) and analyzed using Olympus DP Controller and Management software.

### RNA isolation and RT-PCR Analysis

RNA was purified using RNeasy Mini Kit (Qiagen, Gaithersburg, MD, USA) according to the manufacturer’s instructions. After DNase I (Invitrogen) treatment, cDNA was synthesized by RevertAid First Strand cDNA Synthesis Kit (ThermoFisher Scientific) following the manufacturer’s protocol. Gene expression was measured by RT-qPCR with SYBR Premix Ex Taq II (Takara, Mountain View, CA, USA), using Bio-Rad iQ5 Real-time Thermocycler as described (Bio-Rad, Hercules, CA, USA). Expression levels were determined relative to a standard curve and normalized to GAPDH. Primer sequences are shown in Additional file 6: Table S2.

### Flow cytometry

Cells were fixed with 2 % paraformaldehyde and permeable reagent (eBioscience) for 30 min on ice. Cells were stained with fluorescently labeled antibodies (1 μg/ml; 1 h at room temperature), assayed by flow cytometry (FASCAria II, BD Bioscience, Franklin Lakes, NJ, USA), and data were analyzed by Flowjo7.6 software (Tree Star, Inc. Ashland, OR, USA). Primary antibodies used for staining were anti-human cd71-FITC (eBioscience), mAb to HCV core Antigen (Abcam), and goat anti-mouse Alexa555 antibody (Abcam).

### ELISA

IL-10 and IL-12 concentrations in stimulated cell culture medium were determined by human IL-10 or IL-12 Platinum ELISA kit (eBioscience). Duplicate standard curves were prepared as follows. Briefly, diluted standard proteins were added to plates, incubated at 4 °C overnight, blocked, and antibodies were added. After addition of substrate solution, reactions were stopped by adding 25 μl 2 M H_2_SO_4_. Results were detected by micro-plate reader (BioTek; Shanghai, China) and IL-10 and IL-12 concentrations were calculated against the standard curve by CurveExpert1.3 [19].

### Statistical analysis

One-way ANOVA and Student’s *t*-test were used to determine statistical significance. Analyses were performed with GraphPad Prism Version 5.0 (La Jolla, CA, USA) and SPSS Statistics 21.0 (IBM Corp., Armonk, NY, USA). A *p* value < 0.05 was considered statistically significant.

## Conclusions

In summary, this study demonstrated that HCV antigens impact M1 macrophage polarization. After antigen stimulation, NF-κB signaling is suppressed by up-regulation of the A20/ABIN1 complex, which leads to variation of functional molecules such as CD163, MMPs, TfR1, and CD100, indicating an anti-inflammatory reaction against M1-polarization.

## Additional files

Additional file 1: Figure S1. TfR1 expression level is decreased after HCV antigen stimulation. TfR1 protein expression was suppressed by HCVcc, core, and NS3/4A stimulation by immunofluorescence (A) and cytometry (B). (TIFF 6422 kb) Additional file 2: Figure S4. Transfection efficiency of Ad-A20 and Ad-ABIN1. Transfection efficiencies were determined using signals of GFP co-expression from Ad-A20 and Ad-ABIN1 vectors. The efficiencies were approximately > 90 %. (TIFF 2031 kb) Additional file 3: Figure S2. A20/ABIN1 overexpression reduces HCVcc phagocytosis in M1 macrophages. NS3/4A stimulated macrophages endocytosed fewer HCVcc particles by immunofluorescence (A) and cytometry (B). (TIFF 9199 kb) Additional file 4: Figure S3. A20 and TfR1 affect macrophage phagocytosis activity differently. (A) Gating strategy and phagocytosis activity of macrophage with or without A20 knockdown. Phagocytosis activity of M1 macrophage can be partially rescued by A20 knockdown. (B) Gating strategy and phagocytosis activity of macrophage with or without TfR1 overexpression. No difference was detected in TfR1 overexpressing cells. (Positive cells with TfR1 overexpression vector were selected by FITC marker first; phagocytosis activities are indicated by anti-HCV core in PE channel). (TIFF 16175 kb) Additional file 5: Table S1. Characteristics of the study subjects. (XLS 18 kb) Additional file 6: Table S2 Primers used in the experiment. (XLS 20 kb)
