# Supplementary figures and images for: Up-regulation of A20/ABIN1 contributes to inefficient M1 macrophage polarization during Hepatitis C virus infection
Source: Virol J. 2015 Sep 17;12:147. doi: 10.1186/s12985-015-0379-0 (PMC4574525; doi:10.1186/s12985-015-0379-0)

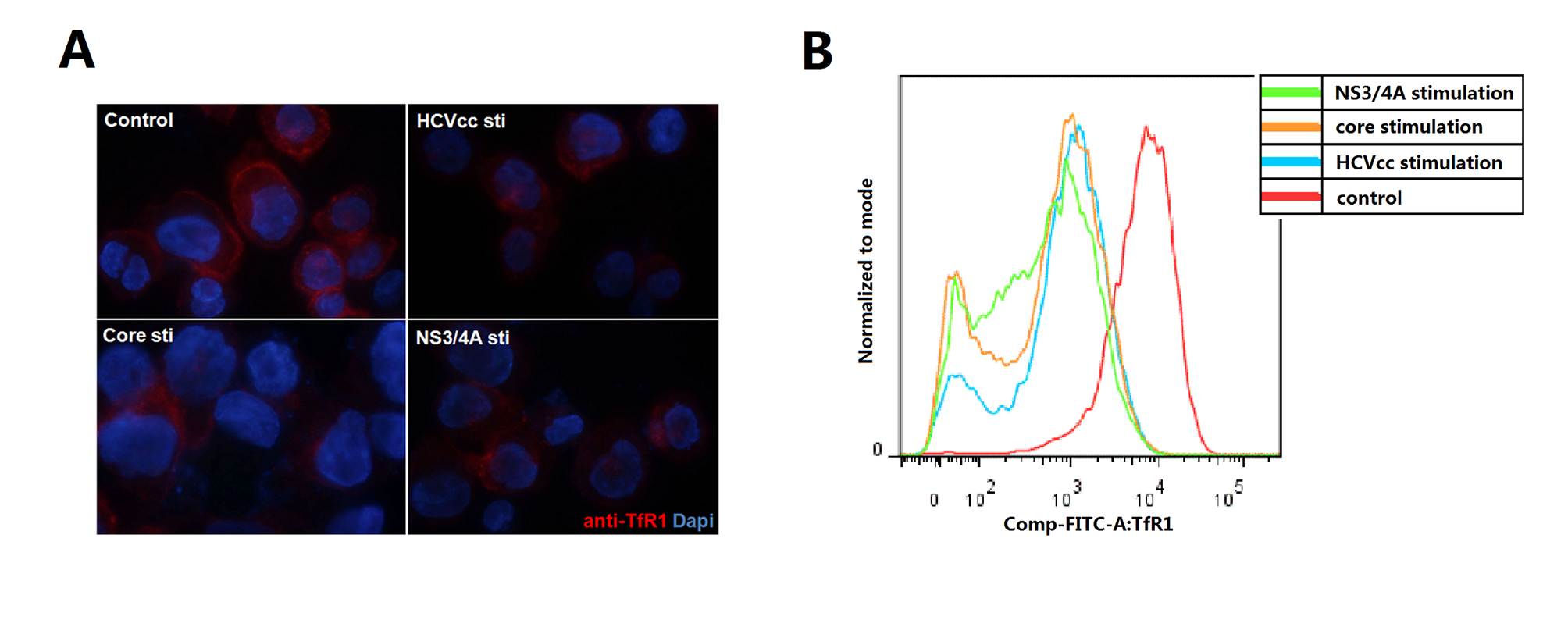

Supplement: Additional file 1: Figure S1. — TfR1 expression level is decreased after HCV antigen stimulation. TfR1 protein expression was suppressed by HCVcc, core, and NS3/4A stimulation by immunofluorescence (A) and cytometry (B). (TIFF 6422 kb) [file 12985_2015_379_MOESM1_ESM.tif]

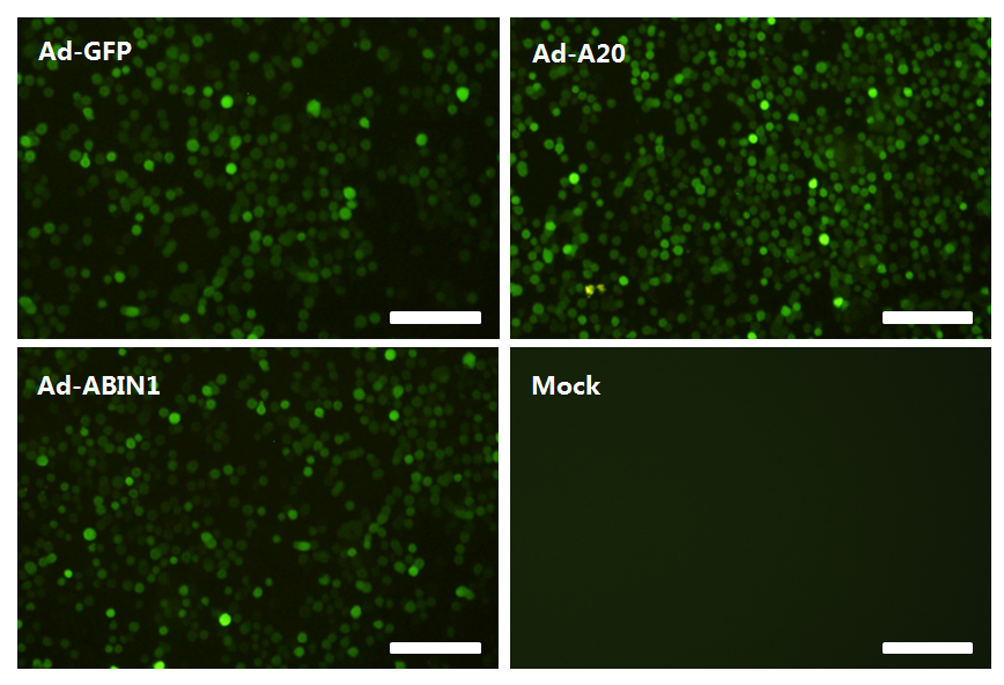

Supplement: Additional file 2: Figure S4. — Transfection efficiency of Ad-A20 and Ad-ABIN1. Transfection efficiencies were determined using signals of GFP co-expression from Ad-A20 and Ad-ABIN1 vectors. The efficiencies were approximately > 90 %. (TIFF 2031 kb) [file 12985_2015_379_MOESM2_ESM.tif]

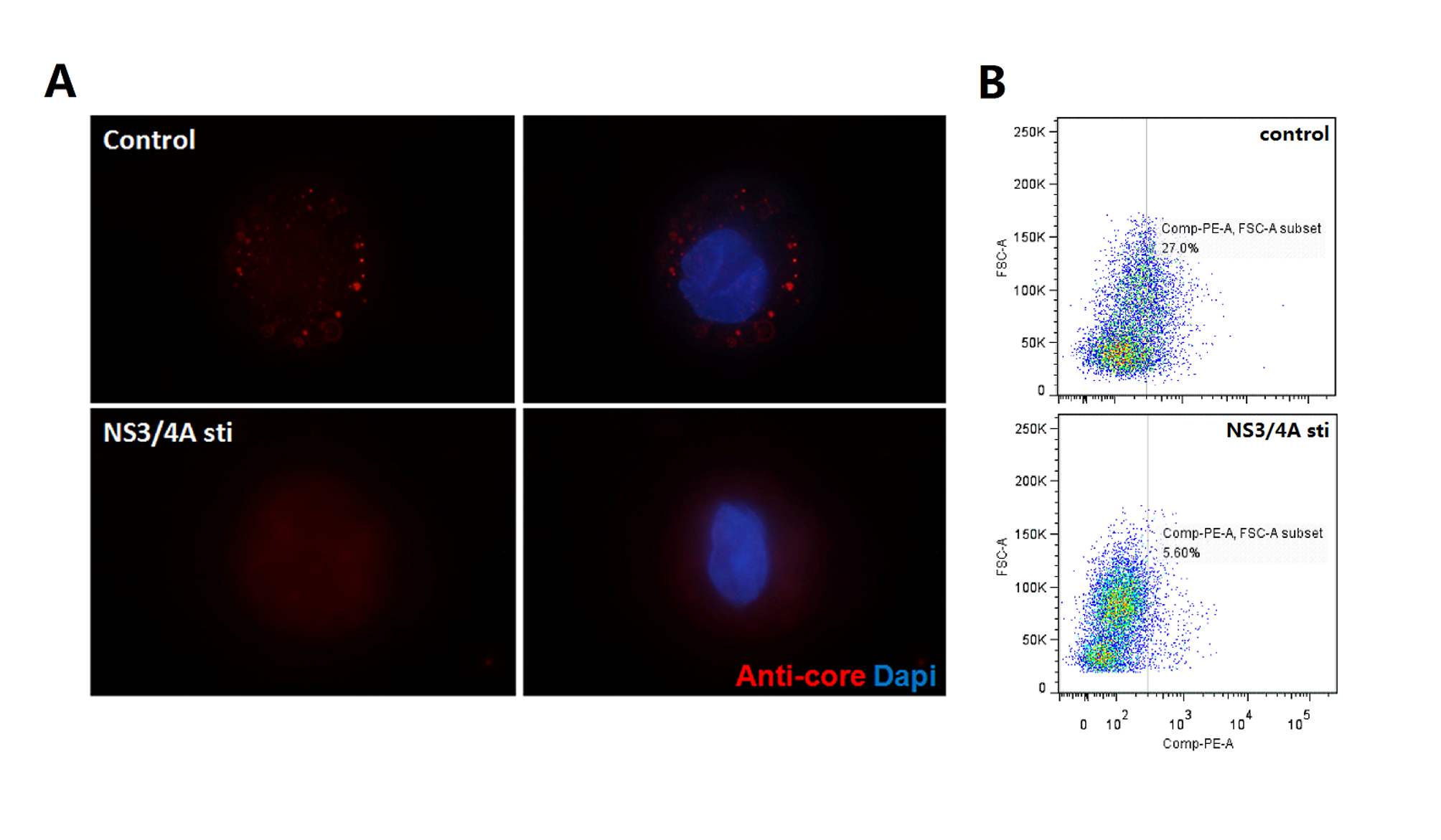

Supplement: Additional file 3: Figure S2. — A20/ABIN1 overexpression reduces HCVcc phagocytosis in M1 macrophages. NS3/4A stimulated macrophages endocytosed fewer HCVcc particles by immunofluorescence (A) and cytometry (B). (TIFF 9199 kb) [file 12985_2015_379_MOESM3_ESM.tif]

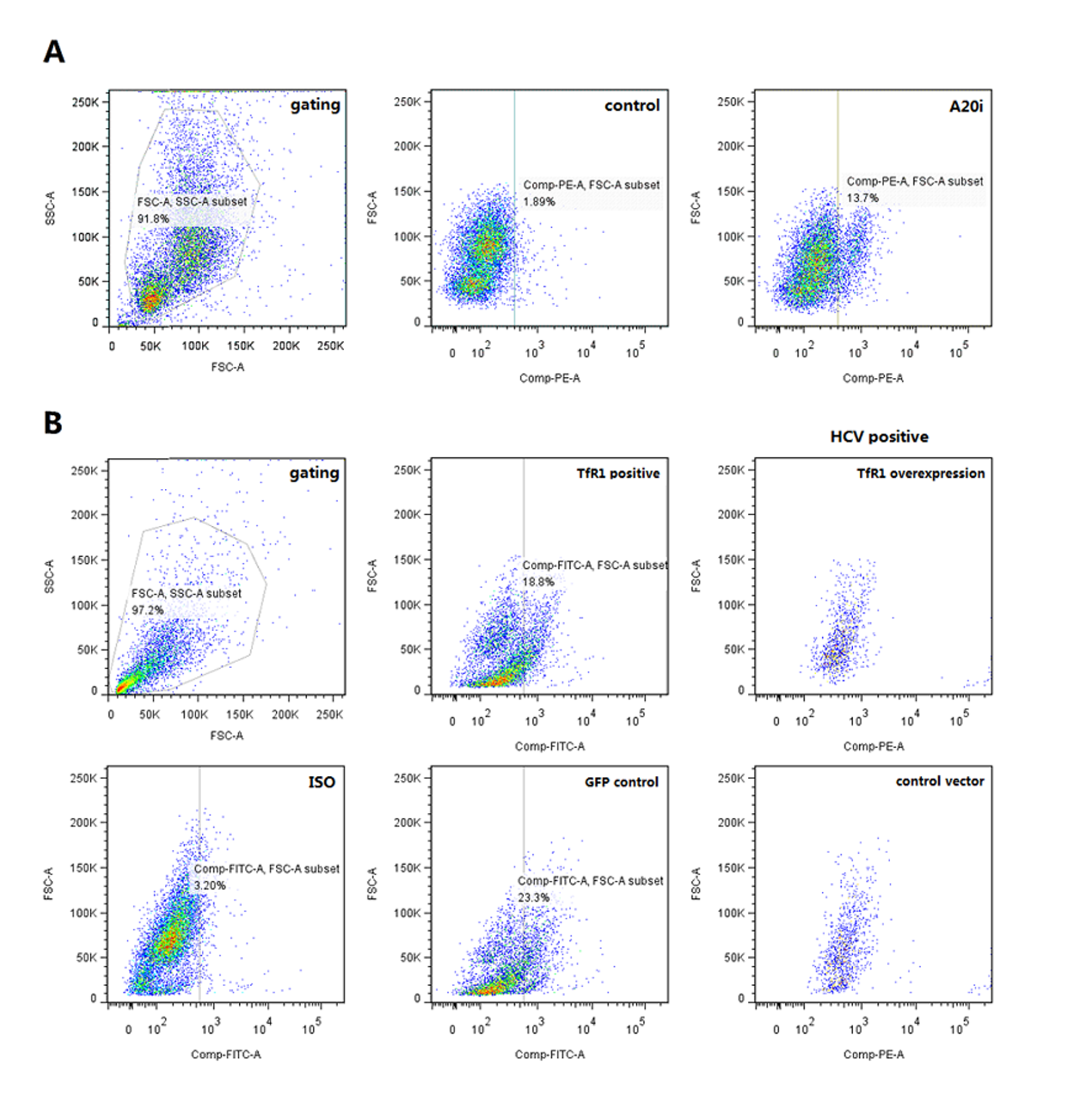

Supplement: Additional file 4: Figure S3. — A20 and TfR1 affect macrophage phagocytosis activity differently. (A) Gating strategy and phagocytosis activity of macrophage with or without A20 knockdown. Phagocytosis activity of M1 macrophage can be partially rescued by A20 knockdown. (B) Gating strategy and phagocytosis activity of macrophage with or without TfR1 overexpression. No difference was detected in TfR1 overexpressing cells. (Positive cells with TfR1 overexpression vector were selected by FITC marker first; phagocytosis activities are indicated by anti-HCV core in PE channel). (TIFF 16175 kb) [file 12985_2015_379_MOESM4_ESM.tif]
